# A Comparative Study of Phenols in Apulian Italian Wines

**DOI:** 10.3390/foods6040024

**Published:** 2017-03-24

**Authors:** Andrea Ragusa, Carla Centonze, Maria E. Grasso, Maria F. Latronico, Pier F. Mastrangelo, Federica Sparascio, Francesco P. Fanizzi, Michele Maffia

**Affiliations:** 1 Department of Engineering for Innovation, University of Salento, via Monteroni, 73100 Lecce, Italy; 2 Department of Biological and Environmental Sciences and Technologies, University of Salento, via Monteroni, 73100 Lecce, Italy; carla_centonze@libero.it (C.C.); nutrizionegrasso@gmail.com (M.E.G.); latronico-francesca@libero.it (M.F.L.); mastrangelo.pf@hotmail.it (P.F.M.); federicasparascio@alice.it (F.S.); fp.fanizzi@unisalento.it (F.P.F.)

**Keywords:** HPLC, wine, polyphenols, antioxidants, hydroxytyrosol, *trans*-resveratrol

## Abstract

Nutraceutics is a growing research field in which researchers study and attempt to improve the biological properties of metabolites in food. Wine is one of the most consumed products in the world and contains a plethora of molecules biologically relevant to human health. In this article, several polyphenols with potential antioxidant activity were measured in wines from Apulia, in Southeast Italy. Hydroxytyrosol, gallic and syringic acids, luteolin, quercetin, and *trans*-resveratrol were identified and quantified by HPLC. The amount of the analyzed metabolites in wines were largely dependent on their color, with red ones being the richest compared to white and rose wines. Gallic acid was the most abundant polyphenol, followed by syringic acid and luteolin. Nevertheless, significant amounts of hydroxytyrosol, quercetin, and *trans*-resveratrol were also found. The average concentration of polyphenols found in these wines could have potential health-promoting effects, especially if consumed in moderate quantities on a regular basis.

## 1. Introduction

Wine is not only one of the most ancient and consumed beverages, but it is also an important product from the nutritional point of view [1]. Among the variety of compounds which determine the overall quality of the final product, polyphenols contribute to several organoleptic properties, such as color, astringency, and flavor [2]. Furthermore, the healthy effect of polyphenols is well-known due to their antioxidant activity and contribute to a variety of biological processes, such as (a) inhibition of the oxidation of low-density lipoprotein (LDL) with a significant effect on atherosclerosis; (b) DNA protection from oxidative stress, with important consequences on the development of some age-related cancers; (c) protection of the vascular system by strengthening capillaries which carry oxygen and other essential nutrients to cells; (d) anti-thrombotic and anti-inflammatory effects; and (e) anti-microbial activity against viruses, bacteria, and hepatotoxins [3,4,5,6]. Polyphenols are produced by the shikimic pathway [7]. However, this path can be found in fungi and bacteria, but it is absent in animals; that is why humans have to introduce phenolic products and aromatic amino acids they need through diet. Phenolic compounds represent, after carbohydrates and acids, the most abundant species in grapes. Nevertheless, the amount and type of polyphenols in wine vary considerably depending on the grape type, environmental factors, production techniques, and conditions [8,9]. The total amount of polyphenols in red wine is usually about 1.5 g/L (80%–90% of which are flavonoids) [6]. Rose wine contains about 400–800 mg/L of polyphenols (40%–60% of which are flavonoids); white wine has only 100–400 mg/L. Depending on the molecular structure and the number of carbon atoms, polyphenols can be roughly grouped into flavonoids, non-flavonoids, and hydroxystilbenes [1]. Flavonoids are found in the peel, seed, and petiole, while non-flavonoid compounds, such as hydroxybenzoic and cinnamic acid derivatives, can be found especially in the pulp. Gallic and syringic acids belong to the hydroxybenzoic acid class and have been shown to induce significant effects on neurodegenerative pathologies, such as Parkinson’s and Alzheimer’s diseases [10,11,12]. Quercetin, part of a subclass of flavonoids called flavonols, has received considerable attention because of its overwhelming presence in foods and its antioxidant activity and is believed to protect against several degenerative diseases by preventing lipid peroxidation [13]. Luteolin, another flavonoid derivative of the flavone subclass, was shown to possess a variety of pharmacological activities, including antioxidant, anti-inflammatory, antimicrobial, and anticancer activities [14]. Hydroxytyrosol is one of the most powerful antioxidants in nature, and extensive research has been carried out trying to exploit its therapeutic effects on the cardiovascular system, neurodegenerative diseases, cancer treatment, osteoporosis, and diabetic neuropathy, to name a few [15,16,17,18,19,20]. The stilbene *trans*-resveratrol has been one of the most extensively studied non-flavonoids due to its well-known health benefits, such as cardioprotective and chemopreventive effects (by inhibiting LDL oxidation) and anti-inflammatory, antibacterial, antifungal, antiviral, neuroprotective, antiproliferative, and anti-angiogenic activities [21,22,23,24,25].

In this article, we measured the above-mentioned polyphenols in several red, white, and rose wines from Apulia and the results were associated with grape type. To the best of our knowledge, few characterization data have been reported in the literature regarding Apulian wines. In pioneering work from the groups of Fanizzi and Sacco, the authors determined several analytes by chemical, chromatographic, and ^1^H NMR techniques in red wines and then managed to identify the origin of the unknown samples by multivariate statistical analysis [26,27,28]. Later, Crupi and colleagues identified and quantified several carotenoid and flavonoid compounds in red and white seedless grape varieties [29,30,31]; Siciliano and co-workers analyzed the volatile components of Negroamaro and Primitivo Apulian red wines [32,33]. Very recently, Barnaba et al. determined the profile of 61 phenols in two of the most common red grapes from Apulia, Primitivo di Manduria and Negroamaro [34]. Nevertheless, to date, quantification and comparison of some of these and other polyphenols in a much wider variety of local red, white, and rose wines has not been reported in the literature.

## 2. Materials and Methods

### 2.1. Chemicals

HPLC-grade reference standard of *trans*-resveratrol was purchased from Dr. Ehrenstorfer GmbH (Augsburg, Germany). Analytical grade gallic acid, hydroxytyrosol, luteolin, quercetin, and syringic acid were purchased from Sigma-Aldrich (Milan, Italy). Acetic acid and HPLC-grade ethanol and methanol were purchased from J.T. Baker (Deventer, The Netherlands). HPLC-grade water was purchased from Carlo Erba Reagenti (Milan, Italy).

### 2.2. Samples

**Figure 1:**
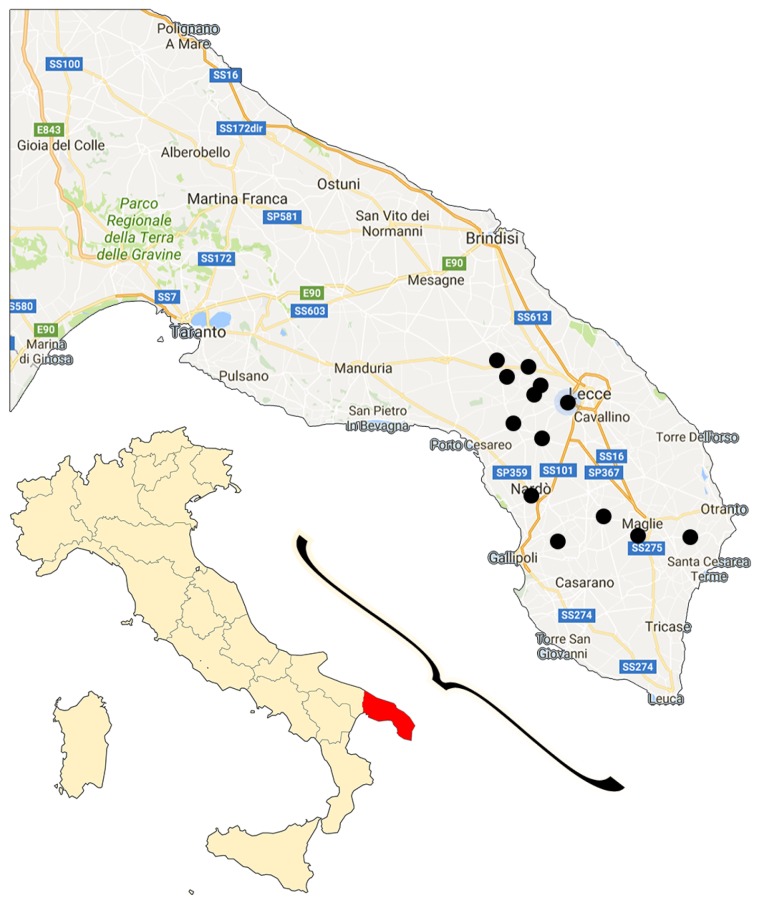
Map of Italy with a zoom on a southern Apulia region (Salento, in red). The black dots represent the sites of production of the wines.

Analysis was conducted on a total of 72 commercially available wine bottles (27 red, 23 rose, and 22 white wines) from Apulia, a region in Southeast Italy (Figure 1). The most characteristic *Vitis vinifera* red grape (Negramaro, Primitivo, and Susumaniello), rose grape (Negramaro, Black Malvasia, and Primitivo), white grape (Bianco d’Alessano, Chardonnay, Falanghina, Fiano, Malvasia, Moscato, and Verdeca), and some combination of these varieties (blend samples) were studied. Details of number of samples, production year, and alcoholic grade for each grape type are given in Table 1. All samples had a denomination of origin (DOC) certification mark, which guarantees the quality and the geographical origin of the wines produced in the Apulia region in Italy.

### 2.3. HPLC Analysis

Separation and identification of phenolic compounds were carried out using an HPLC 1220 Infinity (Agilent Technologies, Palo Alto, CA, USA) interfaced with a diode array detector (model G1315B DAD system; Agilent). A calibration curve was prepared using a standard solution at increasing concentrations of the analytes in ethanol. Fitting of the data was performed through a linear equation with zero intercept (*R*^2^ > 0.98 for all samples). The concentration in mg/kg of the biomolecules in the analyzed samples was calculated through interpolation of the corresponding peak areas. Samples were collected directly from the bottle by a syringe injection through the cork cap. After filtration through a 0.45 µm pore size regenerated cellulose filters (VWR International, Milano, Italy), wine samples were directly injected into an Eclipse Plus C18 (particle size 5 µm; 4.6 × 250 mm, Agilent) stationary phase column. For the detection of *trans*-resveratrol, quercetin, and luteolin, a gradient system of water/methanol/acetic acid (75:20:5, *v*:*v*:*v*) (Solvent A) and water/methanol/acetic acid (50:45:5, *v:v:v*) (Solvent B) was used. The gradient parameters were: 0% B at 0 min, 100% B at 30 min, 0% B at 50 min. The solvent flow was maintained at 1.0 mL/min, the column temperature was set 25 °C, and the UV-Vis detection wavelength was set at 309 nm. Obtained retention times were: *trans*-resveratrol at Min 24.8, quercetin at Min 34.8, and luteolin at Min 42.3. For the detection of gallic acid, hydroxytyrosol, and syringic acid a gradient system of water/methanol/acetic acid (75:20:5, *v:v:v*) (Solvent A) and water/methanol/acetic acid (50:45:5, *v:v:v*) (Solvent B) was used. The gradient parameters were as follows: 0% B at 0 min, 100% B at 40 min, and 0% B at 50 min. The flow was maintained at 1.0 mL/min, the column temperature was set 25 °C, and the UV-Vis detection wavelength was set at 280 nm. Obtained retention times were as follows: gallic acid at Min 3.3, hydroxytyrosol at Min 4.3, and syringic acid at Min 10.1 (The original raw data are available from the Multilab—Chamber of Commerce of Lecce—upon request at multilab@le.camcom.it.).

### 2.4. Statistical Analysis

The amounts of polyphenols reported represent the mean values for a specific wine color and grape type. The reported standard deviation represents the difference among different samples from the same category. If just one sample for a specific grape type was available, no standard deviation has been reported. Standard deviation relatively to replicates of the same sample was always <5%. Obtained polyphenolic values were rounded to one decimal place. Multivariate statistical analysis was performed using STATISTICA 12.5 software (State-Ease Inc., Minneapolis, MN, USA). Principal component analysis (PCA) was performed using the detected polyphenols as variables (*n* = 6) and the wine samples as cases (*n* = 72), yielding a matrix of 432 data points. The analysis was based on correlation and the variances were computed as *SS*/(*N*−1), where *SS* is the Sum of the Squares and *N* is the number of items in the list. In the 2D score plot, cases with sum of cosine^2^ ≥ 0 were reported. General discriminant analysis (GDA) was performed using the type of grape as a dependent variable, the wine color as a categorical predictor variable, and the analyzed polyphenols as continuous predictors.

## 3. Results and Discussion

**Figure 2:**
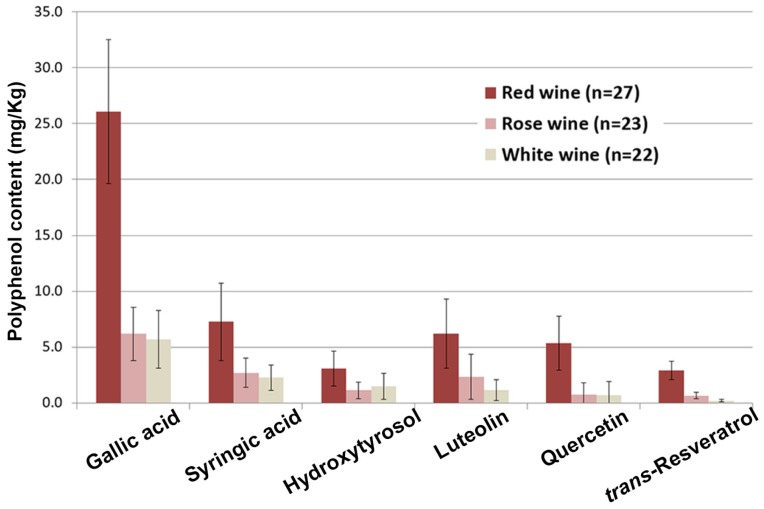
Histogram of the analyzed phenols grouped for wine color.

The content of several polyphenols, namely gallic acid, hydroxytyrosol, luteolin, quercetin, *trans*-resveratrol, and syringic acid, was quantified in different types of commercially available Apulian wines, in Southeast Italy (Figure 1), and the results are shown in Figure 2. The data represent the mean values of the detected polyphenols grouped for wine color. In general, standard deviations are quite high for each data series because of the variability of the samples. In fact, each sample represents a commercially available bottle of a different brand, and even though some of them have the same grape composition, the high variability of all the production parameters led to a quite broad range of data. Nevertheless, all wines were prepared from grapes grown locally and from pure autochthonous varieties, although they were mixed in blend wines, as detailed later in the text. Average production year was about 2012 for red wines and about 2013 for white and rose wines. As expected, alcoholic grade was slightly higher for red wines (average 13.6% ± 0.6%), while white and rose ones presented almost the same value (about 13%).

All the 72 commercially available samples (27 red, 22 white, and 23 rose wine bottles) showed significant amounts of the investigated polyphenols. As expected, red wines contained the highest concentrations, while white and rose wines had less, with rose wines generally presenting slightly higher values compared to white ones.

The results clearly indicate that gallic acid is the most abundant polyphenol in all wines, although there is quite a large difference when comparing their color. In fact, red wines averaged 26 mg/kg, about four times the quantity found in white and rose ones (around 6 mg/kg in both varieties). Considerable amounts of syringic acid, luteolin, and quercetin were also found in the red varieties (about 7, 6, and 5 mg/kg, respectively). Lower quantities of the same phenols were detected in white and rose wines (about 2–3 mg/kg of syringic acid and about 1–2 mg/kg of luteolin for white and rose wines, respectively). Finally, red wines also contained good amounts of hydroxytyrosol and *trans*-resveratrol (about 3 mg/kg each). On the other hand, half that amount of hydroxytyrosol was found in white and rose wines, and even less of *trans*-resveratrol. The results of a more detailed analysis of the quantified polyphenols grouped by type of grape are shown in Table 1.

### 3.1. Red Wines

A total of 27 red wines were analyzed, most of which were Negroamaro (16 samples), followed by Primitivo, blend, and Susumaniello grapes (5, 4, and 2 samples, respectively). The most abundant polyphenol observed was found to be gallic acid (between about 24 and 28 mg/kg in all samples). Lower amounts of syringic acid, luteolin, and quercetin were detected in all wines, with averages of about 7, 6, and 5 mg/kg, respectively (see Table 1 for details). The highest content of hydroxytyrosol was found in Negroamaro grapes, with an average content of 3.4 ± 1.8 mg/kg. Soon after, blend wines were shown to have 2.9 ± 1.0 mg/kg of hydroxytyrosol, not surprising if we consider that these wines were obtained mainly from Negroamaro grapes (percentages from 50% to 80%), and lower amounts were obtained from Malvasia Nera, Primitivo, and Monte Pulciano grapes. Similar concentrations were observed in Susumaniello and Primitivo grapes (about 2–3 mg/kg). 

Finally, Susumaniello wines had the highest concentration of *trans*-resveratrol (4.3 ± 1.5 mg/kg), while Negroamaro, Primitivo, and blend wines averaged about 3 mg/kg, respectable amounts considering the antioxidant strength of this molecule. The results found are in line with values reported in the literature for this grape variety, although Barnaba and colleagues very recently found somewhat higher concentrations of gallic acid and slightly lower ones of hydroxytyrosol in Negroamaro and Primitivo wines from Apulia [34]. Nevertheless, these variations might be related to different cultivation areas and different production years and parameters.

### 3.2. White Wines

White wines were the ones with most grape varieties, with 8 monocultivar samples and 4 blends, mainly composed of different ratios of Malvasia Bianca with Chardonnay, Verdeca, and Trebbiano grapes. Surprisingly, Chardonnay yielded quite high values of hydroxytyrosol (2.3 ± 1.3 mg/kg), almost comparable to those found in red wines. Much lower amounts were found in Fiano, Bianco d’Alessano, Falanghina, Malvasia, and Negroamaro (from about 1.1 to 0.6 mg/kg). Blend wines contained about 1.3 ± 0.9 mg/kg, quite high values although quite variable too, probably because of the different type of grapes and the different mixing percentages used. Gallic acid was the most abundant analyte found. In particular, Moscato grapes yielded 12.5 mg/kg of gallic acid and Fiano about 8 mg/kg, while the remaining varieties yielded between about 3 and 6 mg/kg. Most notably, Moscato grapes were also shown to have the highest alcoholic grade (14.5%), quite high compared to average values in the analyzed white wines (between 12% and 13%). Malvasia grapes yielded the highest content of syringic acid (about 4 mg/kg), although not much higher than the other wines (about 2–3 mg/kg). Even smaller amounts of luteolin were generally detected (about 1 mg/kg if we exclude the only Moscato sample with 4.5 mg/kg). Verdecca showed to have the highest value of *trans*-resveratrol (0.5 mg/kg) among white wines, while the other grapes yielded about 0.1–0.3 mg/kg. Quercetin was detected only in Chardonnay, Malvasia, and Moscato (about 1 mg/kg), while it was present only in traces of the other white grapes.

### 3.3. Rose Wines

Most rose wines were composed of Negroamaro grape, with 18 monocultivar samples and 4 blends in which it was mixed in different percentages with Malvasia Nera. One sample of Primitivo was also present among rose wines. Nevertheless, all rose wines yielded similar results. Gallic acid was again the most abundant polyphenol found (about 6–7 mg/kg). Much smaller amounts of syringic acid were obtained (about 3 mg/kg), and even less of luteolin and hydroxytyrosol (about 2 and 1 mg/kg, respectively). Rose wines also contained higher values of trans-resveratrol compared to white wines (about 0.6–0.8 mg/kg in all samples), although still about a fourth of the average concentration found in red ones.

### 3.4. Statistical Multivariate Analysis

**Figure 3:**
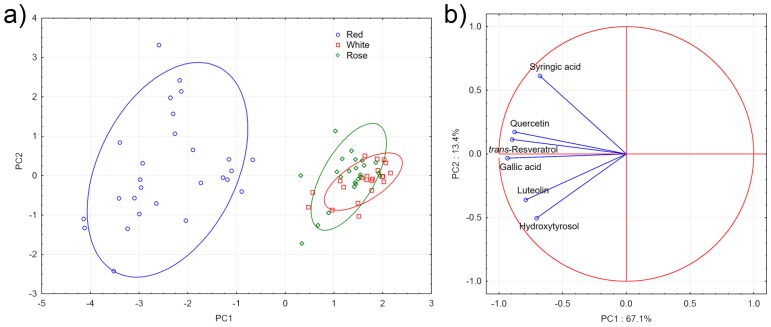
(**a**) Principal component analysis (PCA) scoreplot (PC1 vs. PC2) for red (blue circles), white (red squares), and rose (green diamonds) wines. Ellipses represent the density space of each group with a correlation coefficient of 0.95. (**b**) Loadings plot of PC1 vs. PC2.

Principal component analysis (PCA) was performed using polyphenols data as active variables and wine samples as active cases. Two PCs were extracted: the first one describing 67.1% and the second one 13.4% of the sample variability, respectively. The first two PCs account for 80.5% of the total sample variability. At first glance, it can be seen from the scores plot that red samples spread from about −1 to −4 of PC1, while covering the entire PC2 axis (Figure 3a). 

By plotting the loadings of the variables, it can be seen that gallic acid, *trans*-resveratrol, and quercetin contribute the most to PC1, although not much difference was noted with respect to the contribution (on PC1) of the other variables. Syringic acid, hydroxytyrosol, and luteolin was shown to have a major influence on PC2 (Figure 3b). On the other hand, white and rose wines form two significantly merging groups with positive values along PC1 and average neutral ones along PC2, confirming that these wines in general contain much lower amounts of the detected polyphenols compared to red ones (Figure 3a). The two groups overlap significantly although minor differences can be noted through their density space, drawn by an ellipse with a correlation coefficient of 0.95. The three samples corresponding to the markers at the bottom of the rose wines’ ellipse represent three Negroamaro characterized by a higher hydroxytyrosol concentration. The sample on top of the rose wines’ ellipse corresponds to a Negroamaro sample with a higher concentration of syringic acid. White wines showed even lower variability than rose wines, meaning all samples were quite close to each other in the PCA score plot.

**Figure 4:**
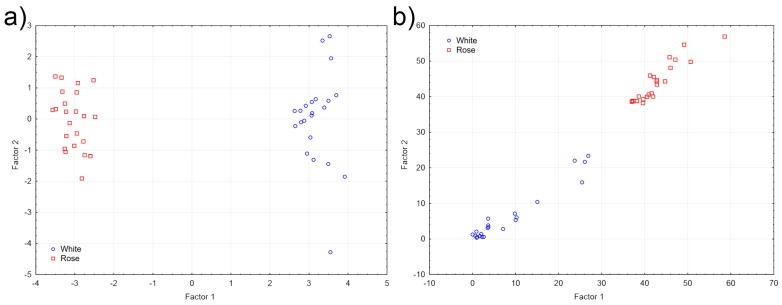
(**a**) GDA scoreplot; (**b**) Mahalanobis distances scatter plot for white (blue circles) and rose (red squares) wines.

A further investigation was performed using a supervised general discriminant analysis (GDA) in order to determine the variables, allowing a better discrimination between rose and white wines. Complete separation between the two groups was obtained, as can be seen in the scatterplot of the correlation scores for Factors 1 and 2 in Figure 4a. *trans*-Resveratrol and, to a minor extent, luteolin was shown to contribute the most to Factor 1, while gallic acid and hydroxytyrosol the most to Factor 2, as suggested by the respective coefficients. Nonetheless, appreciable separation of white and rose wines had already been noticed during the PCA in the scatter matrix plots for all the pairs of variables, in particular when plotting *trans*-resveratrol versus the other variables (data not shown). The discriminatory power of the model toward the two groups was also observed in the Mahalanobis distances scatter plot, as shown in Figure 4b.

## 4. Conclusions

Quantification of biomolecules with nutraceutical properties is of paramount importance, especially nowadays that food quality is being considered more and more. Wine is an aliment with important antioxidant activity due to the presence of a variety of biomolecules, some of which were here characterized by means of HPLC analysis in several commercially available red, white, and rose wines from Apulia, in Southeast Italy. Red grapes were shown to yield the healthiest wines, at least based on the analyzed polyphenols. Gallic acid was found to be the most abundant polyphenol. Nevertheless, red wines also contained high amounts of hydroxytyrosol, especially in those with Negroamaro grapes. High concentrations of hydroxytyrosol were also found in Chardonnay white wines, while the other white and rose grapes analyzed generally contained half those values or even less. Red grapes also produced significant amounts of syringic acid, luteolin, and quercetin. Much lower values of these polyphenols were found in white and rose wines. Red wines yielded good concentrations of *trans*-resveratrol, about 10 times those found in white wines, while rose wines showed an almost intermediate behavior. In general, the amounts of polyphenols found in this study are much lower compared to values used in research studies for curing, e.g., cardiovascular or cancer diseases; nevertheless, ingesting similar amounts of these biomolecules (especially *trans*-resveratrol) on a regular basis is at the origin of the French paradox assumption [35,36,37]. The results here reported were part of a study aiming at characterizing the nutraceutical properties of local Apulian wines, but they can be also exploited to modify the production parameters of these wines to improve their already beneficial properties. Furthermore, these data can be used to determine a metabolic profile of these grapes and can be exploited, in combination with other parameters, to guarantee their traceability and safety when analyzed by multivariate statistical analysis [26,27,28].

## Figures and Tables

**Table 1:** Characteristics and content of phenols in wines organized by type of grape.

Wine Color	Grape Type	*n* of Samples	Year ^1^	Alcoholic Grade ^2^	Gallic Acid	Syringic Acid	Hydroxytyrosol	Luteolin	Quercetin	*trans-*Resveratrol
					Content in mg/kg ^2^					
Red	Blend	4	2010–2013	13.5 ± 0.4	23.7 ± 4.0	7.6 ± 4.2	2.9 ± 1.0	5.7 ± 3.2	5.8 ± 1.3	2.9 ± 0.3
	Negroamaro	16	2007–2013	13.6 ± 0.7	26.8 ± 7.4	7.2 ± 3.4	3.4 ± 1.8	6.3 ± 3.4	5.5 ± 2.7	2.8 ± 0.7
	Primitivo	5	2011–2012	13.6 ± 0.2	24.9 ± 7.0	7.8 ± 4.5	2.5 ± 1.1	6.0 ± 3.2	4.8 ± 3.4	2.8 ± 0.9
	Susumaniello	2	2012	13.3 ± 0.4	28.0 ± 2.2	5.2 ± 3.3	2.7 ± 2.1	7.2 ± 2.4	5.0 ± 1.4	4.3 ± 1.5
White	Bianco d’Alessano	1	2013	12.0	2.7	2.0	0.9	1.1	ND ^3^	0.2
	Blend	4	2012–2013	12.6 ± 1.5	4.5 ± 1.1	2.2 ± 0.6	1.3 ± 0.9	0.8 ± 0.5	0.0 ± 0.0	0.2 ± 0.1
	Chardonnay	9	2012–2013	13.1 ± 0.8	5.5 ± 1.4	2.2 ± 1.7	2.3 ± 1.3	1.3 ± 0.7	1.2 ± 1.8	0.2 ± 0.1
	Falanghina	1	2013	12.5	5.3	2.3	0.8	0.7	ND	0.3
	Fiano	3	2013	13.0 ± 1.0	8.1 ± 4.1	1.8 ± 0.3	1.1 ± 0.3	0.6 ± 0.3	0.3 ± 0.6	0.1 ± 0.1
	Malvasia	1	2013	13.0	4.5	3.6	0.6	1.1	1.0	0.3
	Moscato	1	2012	14.5	12.5	2.0	0.2	4.5	1.0	0.3
	Negroamaro	1	2013	12.5	3.0	2.8	0.6	0.6	ND	0.1
	Verdecca	1	2013	12.5	5.5	2.7	0.4	0.5	ND	0.5
Rose	Negroamaro	18	2011–2013	12.9 ± 0.5	5.9 ± 2.1	2.7 ± 1.4	1.2 ± 0.8	2.3 ± 2.2	0.6 ± 0.8	0.6 ± 0.2
	Negroamaro and Malvasia Nera	4	2013	12.9 ± 0.6	7.4 ± 3.7	2.5 ± 0.7	1.1 ± 0.4	2.4 ± 1.6	1.6 ± 1.8	0.8 ± 0.4
	Primitivo	1	2013	13.5	5.5	3.6	1.1	1.8	ND	0.6

^1^ Production year and alcoholic grade as reported on the bottle. ^2^ Values are expressed as mean ± standard deviation relatively to the different wine bottles analyzed. In some cases only one bottle per grape type was available and no standard deviation was reported. ^3^ Not detected, below the limit of detection.
